# Validation of 3D Knee Kinematics during Gait on Treadmill with an Instrumented Knee Brace

**DOI:** 10.3390/s23041812

**Published:** 2023-02-06

**Authors:** Nicolas Reneaud, Raphaël Zory, Olivier Guérin, Luc Thomas, Serge S. Colson, Pauline Gerus, Frédéric Chorin

**Affiliations:** 1 Université Côte d’Azur, LAMHESS, 06205 Nice, France; 2 Ted Orthopedics, 37 Rue Guibal, 13003 Marseille, France; 3 Université Côte d’Azur, CHU, 06000 Nice, France; 4 Institut Universitaire de France, 75231 Paris, France; 5 Université Côte d’Azur, CNRS, INSERM, IRCAN, 06107 Nice, France

**Keywords:** connected knee brace, inertial measurement unit, joint kinematics, gait, validity

## Abstract

To test a novel instrumented knee brace intended for use as a rehabilitation system, based on inertial measurement units (IMU) to monitor home-based exercises, the device was compared to the gold standard of motion analysis. The purpose was to validate a new calibration method through functional tasks and assessed the value of adding magnetometers for motion analysis. Thirteen healthy young adults performed a 60-second gait test at a comfortable walking speed on a treadmill. Knee kinematics were captured simultaneously, using the instrumented knee brace and an optoelectronic camera system (OCS). The intraclass correlation coefficient (ICC) showed excellent reliability for the three axes of rotation with and without magnetometers, with values ranging between 0.900 and 0.972. Pearson’s r coefficient showed good to excellent correlation for the three axes, with the root mean square error (RMSE) under 3° with the IMUs and slightly higher with the magnetometers. The instrumented knee brace obtained certain clinical parameters, as did the OCS. The instrumented knee brace seems to be a valid tool to assess ambulatory knee kinematics, with an RMSE of <3°, which is sufficient for clinical interpretations. Indeed, this portable system can obtain certain clinical parameters just as well as the gold standard of motion analysis. However, the addition of magnetometers showed no significant advantage in terms of enhancing accuracy.

## 1. Introduction

Knee joint function can be altered by several pathologies. To assess the severity of the effect of these pathologies on locomotion, gait analysis is the gold standard evaluation method for clinicians [1,2]. This type of analysis involves the quantification of the movement of the lower limbs, which characterizes human locomotion [2]. While motion capture with an optoelectronic camera system (OCS) can accurately quantify knee kinematics, such measurements are costly, complex for clinicians, and not ecologically sustainable [3,4]. Recent improvements in this field led to the use of markerless motion capture systems, making it easier to implement. However, this new type of motion capture system is still expensive [5]. Human motion can also be measured with a more affordable system. Placing inertial measurement units (IMUs) on different body segments can accurately quantify three-dimensional kinematics in natural movement situations [6,7]. The measurement system contains a tri-axial accelerometer and a tri-axial gyroscope. The accuracy of this sensor can be improved with the use of a tri-axial magnetometer to limit the drift issue in terms of orientation estimation. This hardware solution entails the use of a fusion algorithm to limit the magnetic field disturbance caused by the proximity of ferromagnetic objects [8].

The three-dimensional kinematics are obtained using the respective orientations in space of each of the magnetic–inertial measurement units (MIMU). The orientation estimation is defined using the Cardan angle with the pitch, roll, and yaw, or with quaternions. Firstly, the gyroscope is used to estimate the orientation of the sensor by integrating the angular velocity. However, this integration method suffers from drift effects. Therefore, the accelerometer is used to estimate the roll and correct the drift on this axis. Secondly, the magnetometer is used to correct the yaw axis by sensing the geomagnetic field. Both the accelerometer and the magnetometer are easily corrupted by external acceleration and magnetic disturbances. To deal with these limitations, sensor fusion has been introduced as a widely accepted method for accurate orientation estimation. Several studies have proposed different methods using a quaternion-based Kalman filter or a complementary filter [9,10]. The low computational cost of a complementary filter with equivalent performance to the Kalman filter methods has led to the popularization of the use of these sensor fusion algorithms for embedded systems [11].

The growing interest in telemedicine and patient monitoring has led to a rapid rise in the use of wearable technologies in healthcare systems [12]. However, a practical approach to monitoring physical rehabilitation or evaluating the physical capacity of a patient at home can be challenging because it is difficult to capture meaningful data in a low-cost and easy-to-use way [13]. The monitoring system must be minimally burdensome for the patient, in order to obtain natural movement data and to ensure high patient acceptance levels regarding the technology. Therefore, quick, easy-to-use calibration is increasingly becoming a benefit for remote patient monitoring [14,15]. 

In this vein, a novel instrumented knee brace was developed, with two MIMUs embedded in a compressive sleeve. These sensors measure the movement of the thigh relative to the shank. The integrated system enabled a quantified analysis of knee motion via a smartphone application, without the need for clinicians, due to its ease of use. Patients were able to put on the instrumented knee brace, carry out calibration movements using the smartphone application with complete autonomy, and thus undertake home-based rehabilitation through explanatory videos and biofeedback in real time. Data measured during the rehabilitation process allowed for a quantified rehabilitation program with clinicians and physiotherapists via success scores and pain-scale charts [16].

However, few studies have focused on the use of wearable sensors from the perspective of self-management [17,18]. Before such technology can be used routinely to measure 3D knee kinematics for gait evaluation at home, the device’s reliability and validity need to be reviewed to compare its performance against the gold standard. In this context, a combination of functional tasks to estimate the axes of the anatomical knee-joint angles is an important aspect [19]. The majority of wearable sensors use a combination of functional tasks or postures, with different levels of accuracy [1]. Quick and easy-to-use calibration tends to be beneficial for remote patient monitoring. Moreover, the improvement in accuracy with better calibration allows for the extraction of the clinical parameters used for monitoring rehabilitation and may be relevant for clinicians [1].

Therefore, the purpose of this study was to evaluate 3D knee kinematics from an instrumented knee brace, calibrated with a new combination of functional tasks, and then compare the performance to an OCS that is calibrated anatomically. The first hypothesis was that the instrumented knee brace could measure 3D kinematics during a gait test with an acceptable level of error (under 5 degrees) in three axes. The second objective was to assess the value of adding magnetometer data in gait-test evaluations. The second hypothesis was that an instrumented knee brace with IMUs and magnetometers would be more accurate.

## 2. Materials and Methods

### 2.1. Subjects

Thirteen healthy young adults (six females, seven males, 25.4 ± 2.2 years, height: 173.4 ± 7.0 cm, body mass: 71.5 ± 17.8 kg) volunteered to participate in the study after providing informed written consent and GDPR consent. They were free of any known disorders that would affect gait and functional mobility. The study was approved by the local ethics committee and was conducted in accordance with the Declaration of Helsinki. 

### 2.2. Materials and Instrumentation

**Figure 1:**
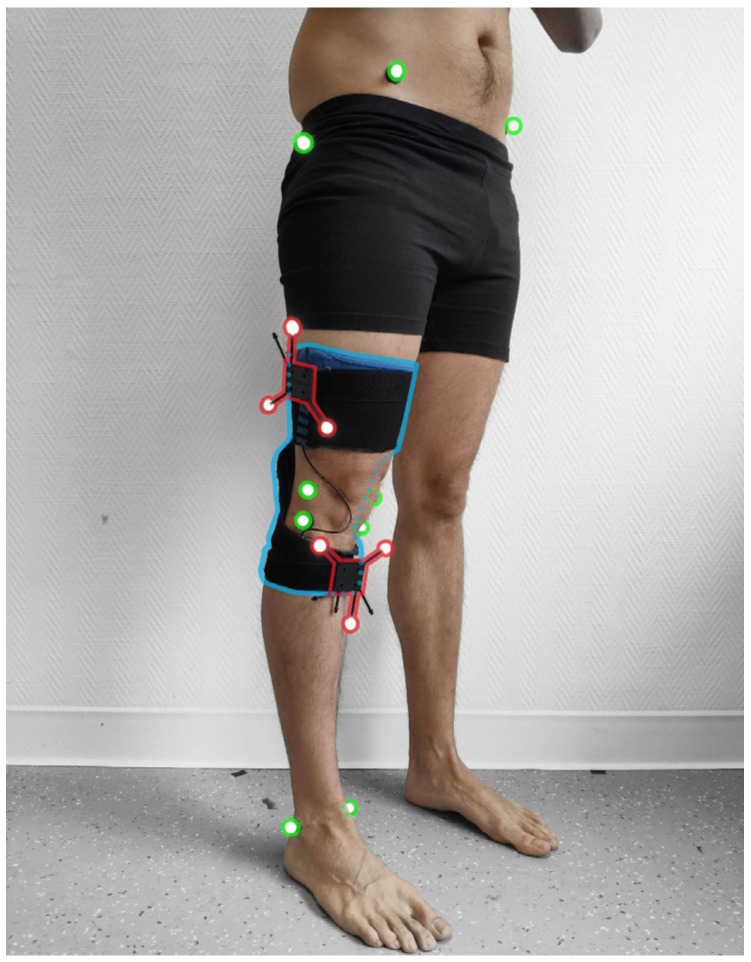
The instrumented knee brace is highlighted in blue, the anatomical reflective markers used for bone calibration are in green, and clusters with reflective markers used for knee kinematics are in red. Anatomical reflective markers were fixed onto the medial malleolus (MM), the lateral malleolus (LM), the medial and lateral epicondyle of the tibia (MT, LT), the medial and lateral epicondyle of the femur (MF, LF), the greater trochanter, the right and left anterior superior iliac spine, and the right and left posterior superior iliac spine.

Knee kinematics were captured simultaneously using the Ted K-Ortho instrumented knee brace (Ted Orthopedics, Marseille, France) on the dominant lower limb in all participants, working with a network of 9 Optitrack Prime 13 optoelectronic cameras (NaturalPoint, Inc., Corvallis, OR, USA). The dominant leg was defined by establishing the preferred leg for dynamic tasks (kicking a ball) [20]. Knee kinematics with the OCS were obtained via two clusters of three reflective markers, held against the lateral side of the thigh and the anterior side of the shank with stretch bands. For knee calibration, ten anatomical landmarks were identified, using reflective markers fixed onto the skin (Figure 1). Knee kinematics with the instrumented knee brace were obtained with the orientation of two ICM-20948 IMUs positioned in a soft compression sleeve, with no mechanical properties except for fixing the sensors to the thigh and shank. The IMU offers 16-bit resolution for accelerometers, gyroscopes, and magnetometers. However, due to Bluetooth low-energy communication requirements, these resolutions were downgraded to 12 bits, with a sensitivity of 1.95 mg/LSB (milli acceleration/least significant bit) for the ±4 g range, a sensitivity of 0.24 dps/LSB (degrees per second/least significant bit) for a range of ±500 dps, + and a sensitivity of 2.39 µT/LSB (micro Tesla/least significant bit) for a range of ±4900 µT, respectively. Kinematic data obtained with the instrumented knee brace and the OCS had a sampling frequency of 100 Hz. 

### 2.3. Protocol

#### 2.3.1. Gait Test

Before the gait test, all participants completed a familiarization period on the treadmill at their comfortable speed. Comfortable gait speeds were measured with a 10 m gait test on an OptoGAIT portable optometric system (Microgate, Bolzono, Italy) in barefoot conditions. The motion capture systems were calibrated after the familiarization period. Each participant performed one 60-second gait test on the treadmill at their own comfortable speed, in barefoot conditions (1.39 ± 0.12 m·s^−1^).

#### 2.3.2. Calibration of Clusters

To evaluate the position of the clusters relative to the bones, the participants completed both a static and a functional calibration procedure. Static anatomical calibration was performed according to standard laboratory protocol. Participants performed a movement with the lower limb fully extended, making a circular hip movement. The hip joint can be modeled as a ball-and-socket joint, and the hip joint center (HJC) location can be characterized by a point that is invariant in any position of the joint [21]. The coordinates of the femoral head location were obtained by an optimization method to minimize the HJC movement during the circular movements, compared with the hip reference [22]. The distal femur location was determined at the middle of the MF and LF markers. The proximal and distal tibia locations were calculated between the MT and LT markers and the MM and LM markers, respectively. 

The mechanical axis of the femur was defined by the line containing the femoral head location and the distal femur location. The femoral condylar axis was defined by the MF and LF markers. The femoral frontal plane was defined as the plane containing the mechanical axis and the femoral condylar axis. The femoral sagittal plane was defined using the femoral axis and the cross-product of the femoral axis and the femoral condylar axis [21]. The mechanical axis of the tibia was defined by the line containing the proximal and distal locations of the tibia. The tibial condylar axis was defined by the MT and LT markers. The tibial frontal plane was defined by the plane containing the mechanical axis and the tibial condylar axis. The tibial sagittal plane was defined using the tibial axis and the cross-product of the tibial axis and the tibial condylar axis [21]. 

#### 2.3.3. Instrumented Knee Brace Calibration

Prior to calibration, the participant must put the brace with the thigh sensor on the lateral face of the thigh and the shank sensor on the anterior-medial surface of the tibia [17]. Two marks indicate the placement of the sensors in the instrumented knee brace. To evaluate the orientation of the IMUs relative to the bones, the participants conducted a static and also a functional calibration procedure. The system was calibrated by having the participant remain stationary in a neutral reference posture, with the lower limb fully extended during calibration. The mechanical axes of the femur and the tibia were defined by the gravity vector. Then, participants performed a functional movement comprising small knee flexions, considering the joint as a hinge joint, when extended, to define the extension/flexion axis [21]. The sagittal plane of the knee was defined by the movement of the IMU of the thigh, compared to the IMU of the shank, during the small flexions. To synchronize the two systems, a cross-correlation between flexion and extension was used to identify the time difference between the OCS and IMU systems.

#### 2.3.4. Sensor Fusion Algorithm

The sensor fusion algorithm used by the instrumented knee brace is a two-step complementary filter [11]. This method decouples the pitch and roll estimation (attitude) from the yaw estimation (heading). The accelerometer and the gyroscope are used to estimate the attitude in quaternion form. This quaternion is used as a correction for the roll and pitch components. Then, the magnetometer data are used for the second step of the complementary filter, to correct the heading of the previous estimation by performing a rotation about the global *z*-axis, in order to align the current frame with the magnetic field. The remainder of this section closely follows the work of Valenti et al. [11].

The orientation in the 3D space of the sensor frame with respect to the earth frame can be represented by a unit quaternion, qES, defined as follows:

(1) qES=q0q1q2q3T=cosα2exsinα2eysinα2ezsinα2T

where α is the rotation angle and *e* is the unit vector that represents the rotation axis.

Unit quaternions can be applied to operate the rotations of 3D vectors. For example, the vector vq S, expressed with respect to the sensor frame, can also be expressed with respect to the earth frame using the following operation:

(2) v Eq=qSE⊗v Sq ⊗ qSE*=qES*⊗v Sq ⊗ qES 

where the vector *v* is expressed as a quaternion:

(3) $$v_{q}=\left[\begin{array}{cc} 0 & v \end{array}\right]{\;}^{T}={\left[\begin{array}{cccc} 0 & v_{x} & v_{y} & v_{z} \end{array}\right]}^{T}.$$

We defined the quaternion obtained with the magnetometer $q_{mag}$ to have only a single degree of freedom, to correct the heading around the *z*-axis only, by setting it to:

(4) $$q_{mag}={\left[\begin{array}{cccc} q_{0_{mag}} & 0 & 0 & q_{3_{mag}} \end{array}\right]}^{T}.$$

The rotation defined in Equation (2) can be expressed in a matrix, as in Equation (5):

(5) v E=RqSEv S

where RqSE is the direct cosine matrix, given in terms of the orientation quaternion qSE, as shown below:

(6) RqSE=q02+q12−q22−q322q1q2−q0q32q1q3+q0q22q1q2+q0q3q02−q12+q22−q322q2q3−q0q12q1q3−q0q22q2q3+q0q1q02−q12−q22+q32.

**Figure 2:**
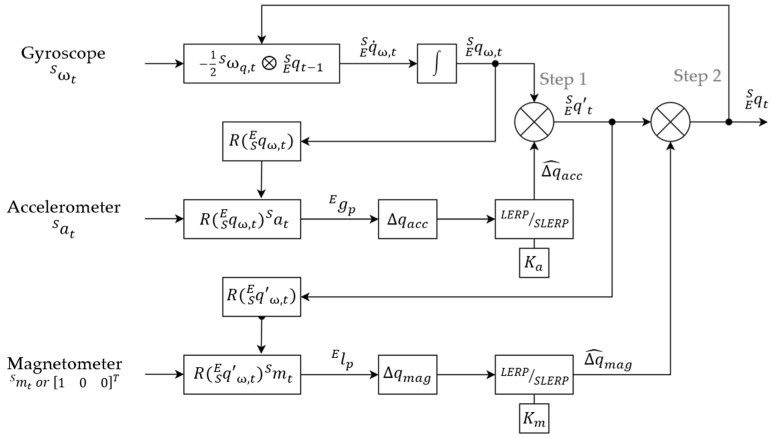
Block diagram of the implemented quaternion-based two-step sensor fusion algorithm, based on the work of Valenti et al. [11]. A canonical vector replaces the magnetometer for the IMU alone. $K_{a}\;$= 0.2 and $K_{m}$ = 0.1; *ω*: measurement from the gyroscope; *a*: measurement from the accelerometer; *m*: measurement from the magnetometer; ${\hat{\Delta q}}_{acc}$: orientation estimated from the accelerometer; ${\hat{\Delta q}}_{mag}$: the correction quaternion, calculated from the magnetometer; qES: the orientation of the sensor frame, relative to the earth frame; ⊗: quaternion multiplication operator.

This two-step complementary filter is based on the work of Valenti et al. [11]. The block diagram of the method is shown in Figure 2. Before the first step for the complementary filter, the gyroscope bias is removed with the calibration file that was obtained through the static phases during the instrumented knee-brace calibration procedure. Then, the complementary filter is initialized with a first estimation of the sensor orientation in quaternion form, with the accelerometer and the magnetometer data. Assuming this initial condition, the filter calculates the quaternion derivative, q˙ESω,t, describing the rate of change of the orientation, ω, obtained through the gyroscope and the previous frame of the sensor [11].

(7) q˙ESω,t=−12ω Sq,t⊗qESt−1

Then, the orientation of the sensor, qESω,t, can be obtained by integrating the quaternion derivative. This first orientation estimation is used in the first step of the complementary filter to predict the gravity vector, g Ep, and calculate the deviation from the real gravity vector, g E, defined as $\Delta q_{acc}$. The delta quaternion, $\Delta q_{acc}$, is then filtered to reduce the noise with a linear interpolation (LERP) or a spherical linear interpolation (SLERP), depending on the deviation of the predicted gravity vector from the real gravity vector, according to the work of Valenti et al. [11]. The LERP is used in the case of small deviation, when ‖sing Ep·g E‖<0.01, using the equation below:

(8) $${\bar{\Delta q}}_{acc}=\left(1-K_{a}\right)q_{I}+K_{a}\Delta q_{acc}$$

where $q_{I}$ is the identity quaternion and $K_{a}\;\in \left[0,\;1\right]$ is the gain that characterizes the level of confidence in the accelerometer, compared to the gyroscope. 

Then, the delta quaternion ${\bar{\Delta q}}_{acc}$ is normalized:

(9) $${\hat{\Delta q}}_{acc}=\frac{{\bar{\Delta q}}_{acc}}{\left\|{\bar{\Delta q}}_{acc}\right\|}\;.$$

If ‖sing Ep·g E‖≥0.01, the SLERP is used to filter the $\Delta q_{acc}$ with the following equation:

(10) Δq^acc=sin(1−Ka∗g Ep·g E)sing Ep·g EqI+sinKa∗g Ep·g Esing Ep·g EΔqacc.

**Figure 3:**
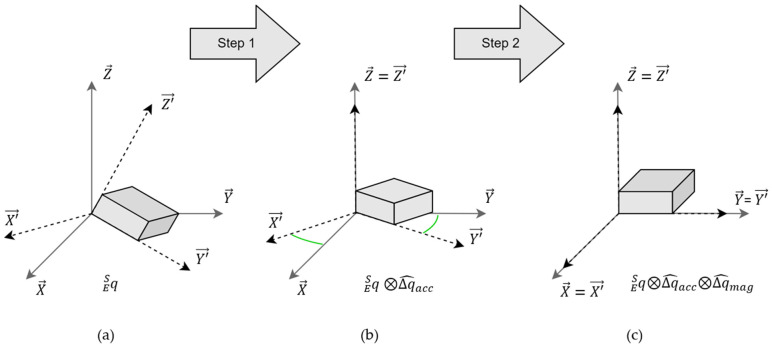
The schematic of orientation estimation-solving with the two-step complementary filter: (**a**) is the initial orientation of the sensor, with the sensor frame $R_{MIMU}=\left(\vec{X^{\prime }},\vec{Y^{\prime }},\vec{Z^{\prime }}\right)$ in the Earth frame $R_{earth}=\left(\vec{X},\vec{Y},\vec{Z}\right)$; (**b**) shows the first correction on the attitude, with the delta quaternion, ${\hat{\Delta q}}_{acc};$ (**c**) shows the second correction on the heading, with the delta quaternion, ${\hat{\Delta q}}_{mag}$.

Finally, the quaternion estimated from the gyroscope data is multiplied by the filtered delta quaternion, to correct the orientation estimation in the attitude component (Figure 3b).

(11) q′ESt=qESω,t⊗Δq^acc

A second step is performed to apply a correction on the heading component. In the same way as the previous step, the magnetometer data is used to compute a delta quaternion between the predicted geomagnetic field, l Ep, and the real one, l E. This delta quaternion, $\Delta q_{mag}$, performs a rotation that is only about the global *z*-axis, by aligning the global *x*-axis into the positive direction of magnetic North [11] (Figure 3c). If the magnetic field data is not provided, a canonical vector on the *x*-axis (Figure 2) replaces the magnetometer vector. The filtered delta quaternion of the magnetometer, ${\hat{\Delta q}}_{mag}$, is calculated with the same method of LERP or SLERP presented previously, with a constant gain, $K_{m}\in \left[0,\;1\right]$ (Figure 2). The second correction of the orientation estimation is obtained by the multiplication of the filtered delta quaternion, ${\hat{\Delta q}}_{mag}$, with the orientation estimation in quaternion form, obtained via step 1 of the complementary filter.

(12) qESt=q′ESt⊗Δq^mag=qESω,t⊗Δq^acc⊗Δq^mag

#### 2.3.5. Knee Kinematics

The kinematics obtained with the instrumented knee brace and the OCS were calculated by the quaternion, qShTh, which defines the orientation of the thigh frame, qETh, relative to the shank frame, qESh.

(13) qShTh=qETh⊗qESh*=qETh⊗qShE

Then, to obtain the flexion/extension, internal/external rotation, and abduction/adduction, the quaternion, qShTh, was converted to Cardan angles (pitch, roll, and yaw) in the local frame, with the following equations:

(14) $$flex=\tan^{-1}\left(\frac{2\left(q_{0}q_{1}+q_{2}q_{3}\right)}{q_{0}{}^{2}-q_{1}{}^{2}-q_{2}{}^{2}+q_{3}{}^{2}}\right)$$

(15) $$abd=\sin^{-1}\left(2\left(q_{0}q_{2}+q_{1}q_{3}\right)\right)$$

(16) $$rot=\tan^{-1}\left(\frac{2\left(q_{0}q_{3}+q_{1}q_{2}\right)}{q_{0}{}^{2}+q_{1}{}^{2}-q_{2}{}^{2}-q_{3}{}^{2}}\right).$$

### 2.4. Data Analysis

The intra-reliability of the instrumented knee brace was determined by the intraclass correlation coefficient (ICC) type (2,1) between participants and gait cycles, based on the range of motion (RoM) from the first 40 strides [23]. ICC was classified as poor (<0.50), moderate (0.50–0.75), good (between 0.75 and 0.90), or excellent (>0.90) [3]. The validity between the instrumented knee brace and the OCS was calculated, based on the complete acquisition of the three knee angles, using the root mean square error (RMSE) computed for the full curve, without offset difference and Pearson’s correlation coefficient. An error of RMSE of <5° was considered excellent, and between 5° and 10° was considered good [6]. Pearson’s coefficient was evaluated as weak (<0.65), moderate (0.65–0.75), good (0.75–0.85), very good (0.85–0.95), or excellent (>0.95) [3].

From the study by Robert-Lachaine et al., twelve clinical parameters were extracted from the three knee rotations, based on normalized gait cycles, to characterize gait (Table 1) [1]. The assumption of normality-distributed data was checked using the Shapiro–Wilk test. The differences between the IMU system and the OCS, and between the IMUs and MIMUs, were calculated by paired *t*-tests.

## 3. Results

### 3.1. Reproducibility of the Kinematic Variables Recorded with the Instrumented Knee Brace

The ICC (95%) for the kinematic range of motion recorded with the instrumented knee brace showed excellent reliability for the three axes of rotation, both with and without magnetometers, with values ranging between 0.900 and 0.972 (Table 2). 

### 3.2. Concurrent Validity of the Instrumented Knee Brace during a Gait Test

**Figure 4:**
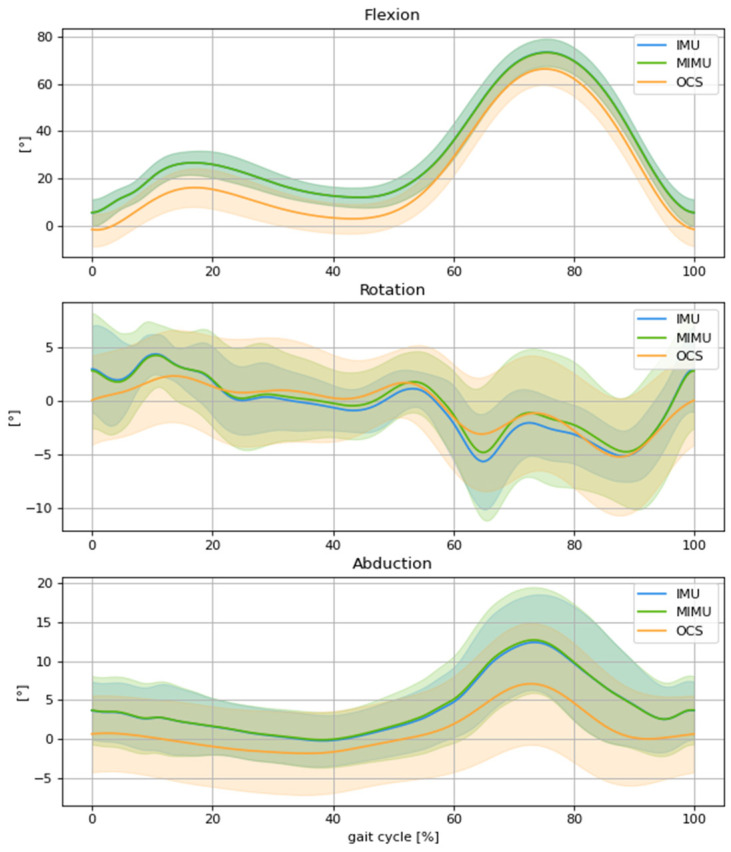
Comparison of mean knee kinematics, normalized to the gait cycle, between the OCS (orange), IMUs (blue), and MIMUs (green), involving flexion, external rotation, and abduction for spontaneous gait speed.

In terms of concurrent validity, Pearson’s r coefficient between systems showed an excellent correlation for the F/E axis (σ > 0.95), a good correlation for the I/E rotation axis (σ > 0.75), and a very good correlation for the A/A axis (σ > 0.85) (Table 2). The RMSE was less than 3° between the instrumented knee brace and the OCS. The addition of the magnetometer data to compute the knee kinematics showed a larger measurement error, especially for the I/E rotation axis, with an RMSE of 3.58°. Paired *t*-tests performed between the IMUs and MIMUs modalities showed a significant difference in the RMSE measured between the instrumented knee brace and the OCS for the tibial rotation and abduction axes (*p*-value < 0.05) (Table 2). The 3D kinematics of the knee are depicted in Figure 4, which compares the measurements with the IMUs, MIMUs, and OCS.

### 3.3. Concurrent Validity of the Gait Parameters Obtained with the Instrumented Knee Brace

Table 3 shows the difference in clinical parameters between the two systems and the two modalities (IMU and MIMU vs. OCS). The instrumented knee brace obtained a similar RoM, with a significantly positive 6° offset for flexion/extension for both modalities (*p*-value = 0.002). The RoM of adduction, the varus thrust, and the tibial rotation were overestimated. Other parameters showed no significant differences between the instrumented knee brace and the OCS (Table 3). 

## 4. Discussion

The purpose of this study was to evaluate the accuracy and reliability of an instrumented knee brace during a gait test, using a new calibration process and with the addition of magnetometers. The results showed excellent reliability and a strong correlation with the OCS. Kinematic data showed a positive offset of 6° for the flexion/extension computed by the instrumented knee brace with the new calibration. However, the first hypothesis was validated, with an acceptable level of error below 5° on the three axes. The second hypothesis was refuted because the study showed a lower accuracy on the abduction and rotation axes with the use of magnetometers, due to the ferromagnetic disturbance caused by the treadmill motors and the metallic structure supporting the harness.

Regarding the validation of the instrumented knee brace, several studies have been conducted to reliably detect knee kinematics with a wearable system. The reliability reported in our study is in line with previous studies, which found fair to excellent intra-rater reliability through a systematic review and, more specifically, ICCs between 0.609 and 0.989, depending on the knee axes [4,6]. This level of reliability is associated with a strong correlation between the gold standard and the instrumented knee brace. The major axis (flexion/extension) showed an excellent correlation, with a lower Pearson’s correlation coefficient, ranging from good to very good, for the minor axes. These results can be explained by a crosstalk effect, which projects the rotations of the sagittal axis onto the frontal and transverse axes, due to small errors in bone orientation estimation during the calibration phases [1,24]. The correlations between systems in the sagittal plane were also similar to those reported in the literature [25,26]. These correlation results were in line with the RMSE values that were obtained with the modalities of the IMUs and the MIMUs, compared to the OCS. The major axis in this study (flexion/extension) showed an RMSE of <2°, while the two minor axes showed a slightly higher RMSE compared with the 5° [17,26] and 3° [4] results reported in previously published studies. Therefore, both systems validate our first hypothesis; the new calibration process provides an acceptable error level under 5°.

Despite this low level of error, the comparison of clinical parameters showed some limitations to the calibration procedure. The results highlighted an offset of 6° on the sagittal axis and led to significant differences for all clinical parameters except the RoM, which is in line with the same overestimation of the initial flexion reported in the study by Kayaalp et al. [17]. Conversely, our study showed a significantly higher RoM in the frontal and transverse planes and no difference for the other parameters, as reported in the study by Robert-Lachaine et al. However, the results of that study are limited because the absolute differences between systems were not reported [1]. These differences in measurements that are highlighted in the current study imply that care should be taken when comparing the gait parameters obtained with the instrumented knee brace to those obtained with another motion capture system.

Based on the RMSE values and *t*-tests between the IMUs and MIMUs, the second hypothesis was rejected, as there was no significant improvement in accuracy in the different axes when incorporating the magnetometers. The results revealed a higher RMSE for abduction and rotation with the addition of magnetometers in the calculation of the kinematics. These findings were in line with those of Robert-Lachaine et al., who suggested that magnetic disturbances might affect the RMSE, specifically the axial axis [8]. The studies of Valenti et al. and Fan et al. explained this issue with a stress test of ferromagnetic disturbances, achieved by placing a magnet near a MIMU sensor during different periods. The results show the immunity of the attitude estimation but a large error in the heading estimation when using the two-step complementary filter [9,11]. This hypothesis was suggested because the steel structure supporting the harness and the motors of the treadmill for the gait test may have induced an inhomogeneous magnetic field during data acquisition [27]. 

A potential improvement of the instrumented knee brace would have been to use an adaptative gain for the magnetometer. The filter presented by Fan et al. showed an adaptative gain, $K_{m},$ for the filter, depending on the magnetic disturbance level. The results demonstrated good performance by using a finite state machine, which switches between different gains depending on the magnetic disturbance level. The measurement error of heading estimation was reduced from more than nineteen degrees to less than one degree [9]. The implementation of this adaptive gain filter for the magnetometer would reduce the error on the heading and may outperform the IMU accuracy of the instrumented knee brace on the axial axis.

A few limitations of this study should be acknowledged when interpreting the results. The OCS was based on clusters and simple kinematics without constraints to obtain the knee angles. This method for calculating kinematics is impacted by soft tissue and skin artifacts (STA) that could introduce measurement errors in the femur and tibia orientations [28]. However, this method was used to compare similar kinematic data and validate the instrumented knee-brace calibration. Therefore, kinematics from the instrumented knee brace showed good axes estimations, but these could be subject to STA, as in our previous study [29]. 

To conclude, a comparison of the instrumented knee brace against OCS measurements during a gait test demonstrated excellent reliability and strong agreement. An IMU-based system seems to be a valid tool by which to assess ambulatory knee kinematics, with an RMSE of <3°, which is sufficient for clinical interpretations. Indeed, this portable system can obtain certain clinical parameters just as well as the gold standard OCS. However, the addition of magnetometers with constant gain showed no significant advantage in terms of enhancing the accuracy with a two-step complementary filter, due to ferromagnetic disturbances in the laboratory. This finding could be different in environments free of magnetic disturbances. 

## Figures and Tables

**Table 1:** Definitions of the calculation of the clinical parameters.

Clinical Parameter	Definition
Initial flexion	Flexion at foot strike
Flexion during loading	Difference between the maximal flexion, from 0% to 20% of the gait cycle, and the initial flexion
Flexion during stance	Difference between the maximal flexion, from 0% to 20% of the gait cycle, and the minimal flexion from 20% to 68% of the gait cycle
Maximal flexion	Maximal flexion of the complete gait cycle
Flexion RoM	Flexion range of motion over the complete gait cycle
Initial adduction	Adduction at foot strike
Varus thrust	Difference between the maximal adduction, from 0% to 20% of the gait cycle, and the initial adduction
Valgus thrust	Difference between the initial adduction and the minimum adduction from 0% to 20% of the gait cycle
Adduction during stance	Mean adduction from 20% to 54% of the gait cycle
Adduction RoM	Adduction range of motion over the complete gait cycle
Initial tibial rotation	Tibial rotation at foot strike
Tibial rotation during loading	Mean tibial rotation from 0% to 20% of the gait cycle
Tibial rotation RoM	Tibial rotation range of motion over the complete gait cycle

**Table 2:** The intraclass reliability and validation of the IMU system. ICC values were assessed on the RoM of the gait cycle. The difference between the IMUs and MIMUs was highlighted by paired *t*-tests (* *p*-value < 0.05).

Data Analysis	Flex/Ext (CI 95%)	Rot Int/Ext (CI 95%)	Abd/Add (CI 95%)
ICC	0.922 (0.850–0.974)	0.923 (0.852–0.974)	0.941 (0.885–0.980)
ICC mag	0.900 (0.821–0.961)	0.901 (0.822–0.962)	0.972 (0.946–0.990)
Pearson’s coefficient	0.997 (0.997–0.998)	0.755 (0.759–0.888)	0.895 (0.653–0.985)
Pearson’s coefficient mag.	0.997 (0.996–0.998)	0.789 (0.747–0.893)	0.873 (0.614–0.972)
RMSE (°)	1.79 (1.61–1.96)	2.16 (1.82–2.50)	2.38 (1.59–3.17)
RMSE mag (°)	1.84 (1.61–2.07)	3.58 (2.00–5.15) *	2.76 (1.64–3.88) *

**Table 3:** Comparison of the clinical parameters. Paired *t*-tests were used and significant *p*-values are identified in bold (*p*-value < 0.05).

Clinical Parameter	OCS	IMU	Δ	*p*	MIMU	Δ	*p*
Initial flexion (°)	−0.4 (7.1)	5.6 (5.9)	−6.0 (1.5)	**0.002**	5.7 (5.9)	−6.0 (1.6)	**0.002**
Flexion during loading (°)	18.0 (6.0)	21.3 (6.6)	−3.3 (0.4)	**<0.001**	21.3 (6.6)	−3.3 (0.4)	**<0.001**
Flexion during stance (°)	14.0 (4.0)	15.8 (4.1)	−1.8 (0.3)	**<0.001**	15.7 (4.1)	−1.7 (0.3)	**<0.001**
Maximal flexion (°)	67.7 (7.1)	73.7 (5.8)	−5.9 (1.4)	**0.001**	73.5 (6.0)	−5.8 (1.4)	**0.001**
Flexion RoM (°)	69.1 (4.9)	68.8 (5.6)	0.2 (0.6)	0.676	68.7 (5.9)	0.4 (0.6)	0.562
Initial adduction (°)	−4.5 (4.9)	−3.6 (3.9)	−0.8 (1.9)	0.676	−3.7 (4.6)	−0.7 (2.0)	0.715
Varus thrust (°)	1.7 (1.2)	2.5 (1.1)	−0.9 (0.3)	**0.016**	2.8 (1.1)	−1.1 (0.3)	**0.017**
Valgus thrust (°)	0.5 (0.6)	0.5 (0.4)	0.0 (0.2)	0.961	0.7 (1.4)	−0.3 (0.4)	0.562
Adduction during stance (°)	−2.6 (4.8)	−0.7 (3.4)	−1.9 (1.5)	0.217	−0.8 (3.6)	−1.8 (1.5)	0.259
Adduction RoM (°)	10.1 (4.0)	14.3 (5.1)	−4.2 (1.4)	**0.011**	15.0 (6.9)	−4.9 (1.8)	**0.020**
Initial tibial rotation (°)	0.0 (4.6)	−3.0 (4.2)	3.1 (1.6)	0.079	−2.9 (5.6)	2.9 (1.7)	0.106
Tibial rotation during loading (°)	−1.6 (4.3)	−3.1 (2.0)	1.4 (1.5)	0.340	−3.0 (3.5)	1.3 (1.5)	0.392
Tibial rotation RoM (°)	11.6 (3.0)	16.2 (5.1)	−4.6 (1.1)	**<0.001**	15.8 (4.8)	−4.3 (1.0)	**<0.001**

## Data Availability

Not applicable.
